# Singular Case of Mortification

**Published:** 1827-12

**Authors:** 

**Affiliations:** St. Thomas's Hospital.


### *Singular Case of Mortification, treated by* Mr. Green, *at* St. Thomas's Hospital.

Edward Younge, ætat. twenty-three, labourer, was admitted into St. Thomas's Hospital, 12th October, 1826, with sloughing of the nose and feet. He is apparently of robust make and healthy constitution. From the description of himself and progress of his complaint, we could learn but little, his senses being far from sound. It appears that he has usually had a good meal a day, and that he has had few opportunities of obtaining spirits. Previous to the appearance of the disease, he slept in the open air for four successive nights, but those nights could not have been attended with frost, being full six weeks before the date of his admission. The disease, he says, began about a month ago, first by coldness in the part, which, gradually turned blue, and was then affected with a burning sensation, after which it wasted away; the surface being of a black colour.

The sore on the face is of a triangular shape, extending from the bridge of the nose to the upper lip, making the nose level with the cheeks, and exposing the alveolar processes of the upper jaw:

there is a dark-brown and dry slough upon the surface, with an evident line of separation, which is furrowed round the sore, showing the ivory appearance of the bone beneath in some places, and just detaching the slough from the edges in the others; there is no blush of inflammation or tenderness. The left foot has a slough upon the toes, similar to the former, though irregularly affecting them, while a livid hue extends up to the ankle. In the right foot, discoloration alone is present. The hands partake also of the same appearance. Each limb is cold to the touch, and produces to the patient the sensation of burning heat. The temperature of the whole body is far below the natural standard; the pulse is 100, and very feeble; tongue dry and brown; great thirst; skin hot; his stools are frequent, and pass almost involuntarily; and his urine is scanty.

Mist. Creta f℥j.; Conf. Arom. ℈j.; Tinct. Opii m. xx. quartâ quâque horâ.—Aquæ Vitæ et Aquæ Tepid. āā ℥ss. omni semihora.—Cat. Lini to the sloughs. Flannel to the discoloured parts.

Nine P.M.—Skin burning hot and dry; pulse sharp and quick; thirst still great.

Brandy every hour only, with double quantity of water.

Eleven P.M.—General excitement the same; the skin hotter.

Brandy every hour and a half, with hot water every half-hour.

13th, seven A.M.—Skin getting cold again, and pulse sunk, but no perspiration.

Brandy every hour.

Twelve o'clock.—Much the same.

℞. Mist. Cretæ f℥ss.; Conf. Arom. ℈j.; Tinct. Opii m. xx. quartâ quâque horâ.—Beef-tea.

14th.—Very little alteration. Diarrhœa less; spirits better, and answers more freely, but there is always a dulness about him.

℞. Sulph. Quinin. gr. v.; Tinct. Opii m. x.; Inf. Catechu ℥iss. quartâ quâque horâ.

Half-past ten P.M.—Delirious, with great restlessness, and tossing about for the last hour, having complained of considerable pain in the affected limbs.

Pil. Opii gr. iij. st. s. et rep^r j. omni semihorâ.

The delirium continued to increase till half-past twelve, when he suddenly expired.

18th.—On examination, nothing unusual presented itself. The liver and kidneys were much enlarged, but their structure, as well as that of every other viscus, was perfectly healthy. The lungs, heart, and large vessels, showed nothing unnatural. A little water was found at the base of the brain.

In this case, the most remarkable circumstance is the obscurity of the cause, in the search of which, neither the history from the patient while living, nor the post-mortem examination, could assist us. Nourishment had been supplied to him in sufficiency, and he could not have been exposed to any great degree of cold. The large arteries indicated no diseased alteration; and we were not induced to examine the smaller vessels, as the same appearances would, in all probability, have been seen as in the large ones.

While in the hospital, his different limbs exhibited all the various stages of the disease very remarkably at once. His hands were slightly discoloured, numbed, and cold to the touch; the right foot was of a deeper cast; and the left exhibited a slough, not circumscribed, but inclined to spread, the black and livid colours being intimately blended together; while on the nose was not only a line of demarkation, but in some parts actual separ ation.

